# Lusutrombopag increases hematocytes in a compensated liver cirrhosis patient

**DOI:** 10.1007/s12328-017-0735-2

**Published:** 2017-03-21

**Authors:** Akira Sakamaki, Takayuki Watanabe, Satoshi Abe, Kenya Kamimura, Atsunori Tsuchiya, Masaaki Takamura, Hirokazu Kawai, Satoshi Yamagiwa, Shuji Terai

**Affiliations:** Division of Gastroenterology and Hepatology, Graduate School of Medical and Dental Sciences, Niigata University, 1-757 Asahimachido-ri, Chuo-ku, Niigata, 951-8510 Japan

**Keywords:** Lusutrombopag, Thrombocytopenia, Liver cirrhosis, Thrombopoietin

## Abstract

A 56-year-old Japanese man with liver cirrhosis (LC) due to hepatitis C virus was admitted to our hospital for radiofrequency ablation of residual tumor following lusutrombopag administration. Laboratory tests revealed thrombocytopenia and leukopenia. The patient’s LC was managed, and he was classified as Child–Pugh A. After admission, lusutrombopag was administered for 7 days. The platelet count increased to over 50,000/mm^3^ after 7–14 days and returned to previous levels 50 days after administration. Leukocyte and erythrocyte counts also increased in response to the treatment and stayed elevated for over 120 days. Lusutrombopag acts selectively on human thrombopoietin (TPO) receptors and activates signaling pathways that promote the proliferation and differentiation of bone marrow progenitor cells into megakaryocytes, consequently increasing the blood platelet count. However, the patient treated with lusutrombopag in our case study showed increased blood leukocyte and erythrocyte counts as well. Given that TPO receptors are reportedly expressed in not only megakaryocyte progenitor cells but also hematopoietic progenitors, lusutrombopag may potentially improve pancytopenia caused by LC and can be used for the recovery of blood counts before other treatments.

## Introduction

Patients with liver cirrhosis (LC) show a marked decrease in blood platelet counts [1], whilst treatment often involves invasive therapies which can decrease the count further. Lusutrombopag, used for 1 week before invasive treatments, increases blood platelet counts in patients with LC and thrombocytopenia, thereby improving the safety of these procedures [2]. On the other hand, not only do platelets decrease but erythrocytes and leukocytes also lower in patients with LC [3], which can interrupt therapies such as chemotherapy and radiation. The mechanism of cytopenia in patients with LC involves two reported factors: hypersplenism and decreased thrombopoietin (TPO) [4]. Serum TPO levels correlate with liver function: serum albumin levels and the Child-Pugh score [5]. TPO is a potent endogenous cytokine that acts through TPO receptors, known as c-Mpl receptors, which are primarily expressed on platelets, megakaryocytes (MKs), and hematopoietic stem cells (HSCs) [6, 7]. We herein report a case of a patient with LC whose whole blood cell counts increased by lusutrombopag administration. Written informed consent for the publication of this case report was obtained from the patient.

## Case report

In August 2015, a 56-year-old Japanese man presented at our hospital for the management of LC complications and was treated for hypertension. After a mass screening revealed liver dysfunction, he was diagnosed with LC with serotype 2 hepatitis C virus (HCV) in February 2015. Following hypertension treatment, he developed additional complications of esophageal varices (EVs) and was admitted to our hospital for further treatment. Endoscopic injection sclerotherapy (EIS) was performed for the EVs in September 2015, and antiviral therapy using a combination of sofosbuvir and ribavirin was initiated in November 2015 for HCV infection; a sustained virological response was achieved. In March 2016, he was admitted for EV rupture, and endoscopic variceal ligation was performed during emergency hospitalization. Moreover, dynamic computed tomography revealed a 20-mm mass with hypervascularity in the arterial phase and washout in the portal phase of segment 8, which led to the diagnosis of hepatocellular carcinoma (HCC). Transcatheter arterial infusion therapy using cisplatin and miliplatin was performed in April 2016; however, residual tumor was detected. The patient was scheduled for radiofrequency ablation (RFA) following lusutrombopag administration and was admitted to our hospital.

A physical examination revealed no jaundice, flapping tremor, and ascites. Laboratory tests revealed thrombocytopenia and leukopenia (blood platelet count, 33,000/mm^3^; leukocyte count, 1090/mm^3^), but erythrocyte counts were within the normal range (416 × 10^4^/mm^3^). Blast cells were not detected in the peripheral blood. Transaminase levels were not increased (aspartate aminotransferase, 20 IU/l; alanine aminotransferase, 18 IU/l) whilst iron overload was also not observed (serum iron, 35 µg/ml; serum ferritin, 10 ng/ml). The tumor marker α-fetoprotein slightly increased (61 ng/ml), but des-gamma carboxyprothrombin was not elevated (27.8 mAU/ml) (Table 1). The patient’s LC was managed, and he was classified as Child–Pugh A (previous Child–Pugh score was 6) with a model for end-stage liver disease score of 4.

**Table 1:** Laboratory data on admission

Hematology	
Leukocyte count (/mm^3^)	1090
Erythrocyte count (×10^4^/mm^3^)	416
Hemoglobin (g/dl)	11.8
Platelet count (mm^3^)	33,000
Coagulation test	
Prothrombin time (%)	60
PT-INR	1.29
Tumor marker	
α-Fetoprotein (ng/ml)	61
AFP-L3 (%)	1.5
DCP (mAU/ml)	27.8
Biochemistry	
Total protein (g/ dl)	7.0
Albumin (g/dl)	3.7
Serum sodium (mEq/l)	140
Serum potassium (mEq/1)	4.0
Serum chloride (mEq/1)	109
Serum iron (μg/ml)	35
Serum ferritin (ng/dl)	10
Total bilirubin (mg/dl)	1.0
Direct bilirubin (mg/dl)	0.2
AST (IU/1)	20
ALT (IU/1)	18
LDH (IU/1)	119
ALP (IU/1)	205
GGT (IU/1)	37
Blood urea nitrogen (mg/dl)	14.0
Creatinine (mg/dl)	0.6
C-reactive protein (mg/dl)	0.03

*PT-INR* international normalized ratio of prothrombin time, *AFP-L3* lens culinaris agglutinin a-reactive α-fetoprotein, *DCP* des-gamma carboxyprothrombin, *AST* aspartate aminotransferase, *ALT* alanine aminotransferase, *LDH* lactate dehydrogenase, *ALP* alkaline phosphatase, *GGT* gamma-glutamyl transpeptidase

**Fig. 1:**
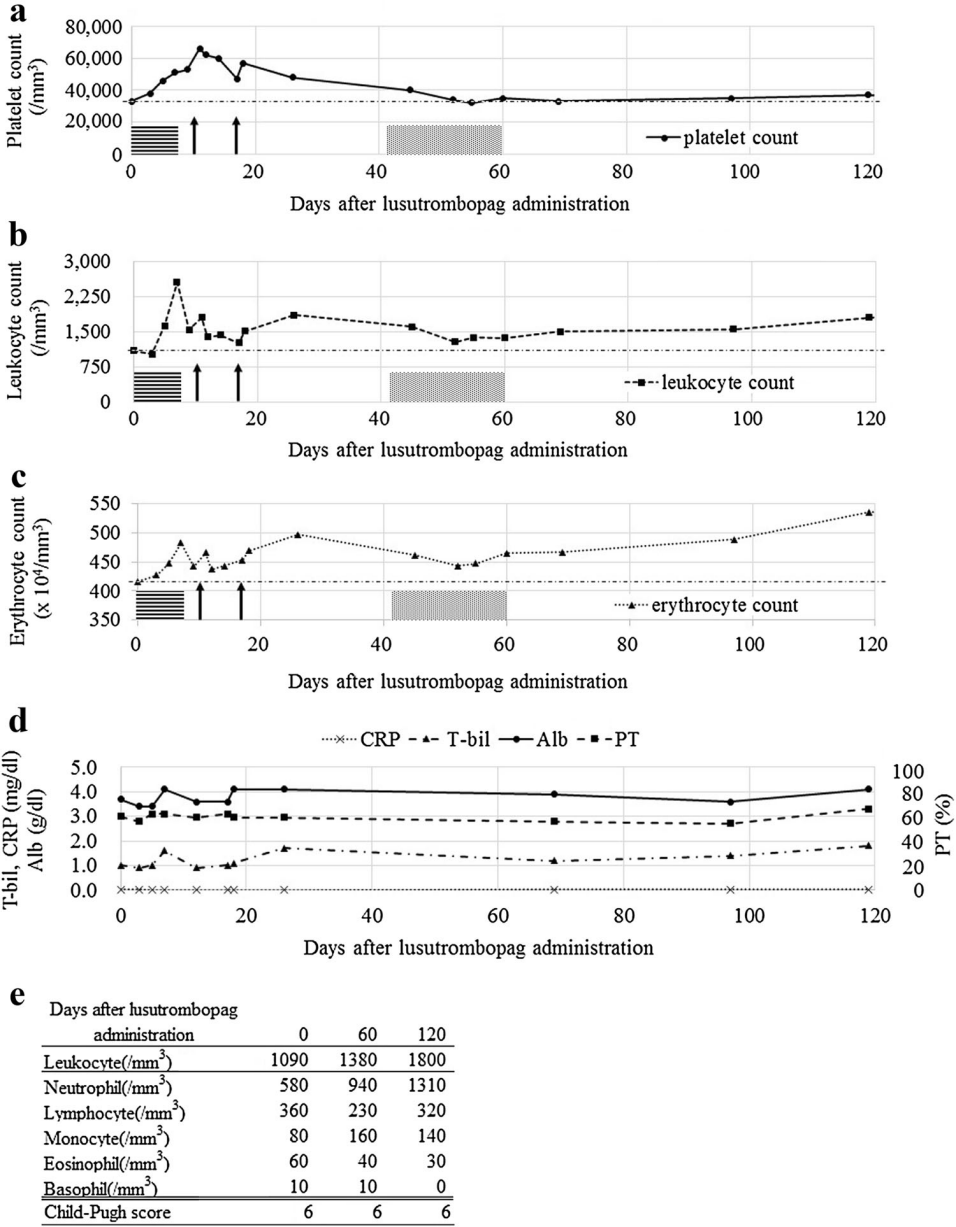
The hematocyte counts after lusutrombopag administration. The platelet counts (**a**) increased to over 50,000/mm^3^ 7–14 days and returned to the previous levels 50 days after lusutrombopag administration. Leukocyte (**b**) and erythrocyte (**c**) counts also increased in response to the treatment and stayed at high levels for over 120 days. Serum albumin, bilirubin, and prothrombin time, and C-reactive protein levels remained invariant; the reduction of liver function and inflammation reaction were not observed (**d**). The differential counts of leukocytes indicated the increase of neutrophil and monocyte counts, however, there was no increase of eosinophil, basophil and lymphocyte counts. In addition, the Child-Pugh score also remained invariant (**e**). *Horizontal line boxes* lusutrombopag administration; *black arrows* tried radiofrequency ablation; *meshed pattern boxes* conventional radiation therapy; *chain line* the hematocyte levels before lusutrombopag administration. *CRP* C-reactive protein, *T-bil* total bilirubin, *Alb* albumin, *PT* prothrombin time

After admission, lusutrombopag was administered for 7 days. The platelet count increased to over 50,000/mm^3^ 7–14 days after administration (Fig. 1a). RFA was scheduled 10 and 17 days after starting lusutrombopag but was suspended because the HCC lesion was no longer detected by ultrasonography. Conventional radiation therapy was initiated for residual tumor tissue. Blood platelet counts returned to the previous levels 50 days after lusutrombopag administration. Leukocyte and erythrocyte counts also increased in response to the treatment and stayed elevated for over 120 days (Fig. 1b, c). Furthermore, leukocyte differential counts indicated an increase in neutrophil and monocyte counts but not eosinophil, basophil, and lymphocyte counts (Fig. 1e). On the other hand, liver function (serum albumin, bilirubin, and prothrombin time) and inflammatory marker (C-reactive protein) levels remained invariant (Fig. 1d). In addition, thoracoabdominal-pelvic CT indicated LC change, therapeutic change after radiation, and mild ascites without any recurrence or other diseases over 120 days after lusutrombopag administration.

## Discussion

Early stage HCC patients have an indication for curative treatment including resection, liver transplantation, and RFA. It is important that the patient is indicated for curative treatments or not, but RFA cannot be performed due to liver hemorrhage for patients who have thrombocytopenia. Reports published on a number of cases define the indication for RFA as patients with a blood platelet count over 50,000/mm^3^ [8] or 40,000/mm^3^ [9]. Lusutrombopag acts selectively on human TPO receptors (c-Mpl) and activates signaling pathways that promote the proliferation and differentiation of bone marrow progenitor cells into megakaryocytes, consequently increasing the blood platelet count [6]. In a phase III trial conducted in Japan, 77.1% of the study participants responded to lusutrombopag treatment, with response being defined as the recovery of blood platelet counts to over 50,000/mm^3^ [2].

In some patients, lusutrombopag may cause thrombosis in systemic vessels, such as the portal vein. Eltrombopag, another TPO receptor agonist that is used to treat idiopathic thrombocytopenic purpura, induced thromboembolic adverse events in 2.3% (6/261) of patients with chronic liver disease [10]. These cases reported very high platelet levels (>100,000/mm^3^), suggesting the need for careful follow-up to ensure that platelet levels do not increase too much. Thromboembolic adverse events were seen in 2.1% (1/48) of patients for both lusutrombopag-treated and placebo groups in a phase III trial. Thrombotic risk factors, such as past thrombosis, antiphospholipid antibody syndrome, and prolonged immobility, were also reported to induce TPO receptor agonist-associated thrombosis. Thus, these agents need to be used more carefully in patients with risk factors. Because of the technical difficulties of RFA, invasive therapy was not performed in our case. However, the platelet count increased to over 50,000/mm^3^, and no thrombotic events were observed; thus, lusutrombopag treatment was both safe and effective.

Interestingly, in our case study, the patient treated with lusutrombopag showed increased blood leukocyte (especially neutrophil and monocyte) and erythrocyte counts as well (Fig. 1a–c). Megakaryopoiesis is a complex process that involves the commitment of HSCs to the MK lineage, MK maturation, and terminal differentiation that produces platelets. HSCs are multipotent cells that can either self-renew or differentiate into various blood lineages [11]. One limitation of this study is that we were unable to show reproducibility; although leukocytosis and erythrocytosis were not described in a phase II and III trial for lusutrombopag, another TPO agonist, eltrombopag, improved hematopoiesis including hemoglobin and neutrophil counts in patients with aplastic anemia [12]. In this report, most of the cases involved continuous administration, but one case was discontinued because of side effects, although a long-term increase of neutrophil count was observed. Larisa et al. reported a TPO agonist induces self-renewing HSCs in mice [13]. In addition, Kuter et al. reported TPO is a physiological factor mediating the feedback loop between circulating platelets and bone marrow MKs [14]. TPO agonists increase platelets stimulating MKs, and leukocytes and erythrocytes stimulating HSCs [7, 11]; self-renewing HSCs may cause a long-term increase, but the negative feedback of MKs may lead to the differentiation of HSCs to leukocytes and erythrocytes. In addition, TPO stimulation resulted in tyrosine phosphorylation of the TPO receptor and the common beta subunit of the granulocyte–macrophage colony-stimulating factor (GM-CSF) receptor in the TPO-dependent human erythroleukemia cell line, TF-1/TPO [15]. The hematocyte counts in these reports correspond to those in our case. Furthermore, a hematological disorder could not be excluded completely because no bone-marrow biopsy was performed, although no findings of iron overload or blast cells in peripheral blood indicated cytopenia in this case due to the LC.

More cases will need to be analyzed to determine whether lusutrombopag may potentially improve pancytopenia caused by LC, and whether it can be used for the recovery of blood counts before other treatments, such as chemotherapy.

In conclusion, we present a case wherein the whole blood cell count increased after lusutrombopag administration. Although the mechanism of this increase remains unclear, the accumulation of such cases may lead to new uses of lusutrombopag in the future.
